# Temporary Protective Shoulder Implants for Revision Surgery with Bone Glenoid Grafting

**DOI:** 10.3390/ma15186457

**Published:** 2022-09-17

**Authors:** Daniel Schaffarzick, Karl Entacher, Dietmar Rafolt, Peter Schuller-Götzburg

**Affiliations:** 1 ECS Schaffarzick—Engineering/Consulting/Service, Sankt-Peter-Straße 15/2, A-5061 Elsbethen, Austria; 2 Department of Information Technology and Systems Management, Salzburg University of Applied Science, Urstein Süd 1, A-5412 Puch, Austria; 3 Center for Medical Physics and Biomedical Engineering, Medical University of Vienna, Vienna General Hospital, Währinger Gürtel 18-20, A-1090 Wien, Austria; 4 Department of Prosthetic Dentistry, University Dental Clinic Vienna, Sensengasse 2a, A-1090 Vienna, Austria

**Keywords:** glenoid implant, implant development, finite element analysis, 3D modelling, abrasion test, glenoid defect

## Abstract

This article describes the development of a temporary protective glenoid prosthesis placed between the augmentation and humeral head prosthesis during the healing phase of shoulder prosthesis revision with necessary reconstruction of the bony structure of the glenoid. The glenoid protection prosthesis ensures the fixation of the augmentation material and protects the screws from contact with the metallic humeral head prosthesis. Another approach of the development is a reduction of the resorption of the augmentation by targeted mechanical stimulation of the tissue. The aim should be to achieve significantly improved conditions for the implantation of a new glenoid component at the end of the healing phase of the augmentation material in comparison to the current standard method. The development of the protective prosthesis was carried out according to specific needs and includes the collection of requirements and boundary conditions, the design and technical detailing of the implant, the verification of the development results as well as the validation of the design. For the verification, FEM simulations (Finite Element Analysis) were performed to estimate the mechanical stability in advance. Mechanical tests to confirm the stability and abrasion behavior have been carried out and confirm the suitability of the protective implant. The result of the present work is the detailed technical design of two variants of a glenoid protective prosthesis “GlenoProtect” for use in revision procedures on shoulder joints—with large-volume defects on the glenoid—treated by arthroplasty and the necessity of augmenting the glenoid, including a description of the surgical procedure for implantation.

## 1. Introduction

The annual number of shoulder arthroplasties increases continuously. In 2017, an estimated number of about 800,000 patients were living in the United States with a shoulder replacement with a prevalence of 0.258%, increasing markedly from 1995 (0.031%) and 2005 (0.083%) [1]. In 2008, 27,000 shoulder arthroplasties (total endoprostheses only) were performed [2]. Although the number of shoulder arthroplasties performed is still far below that of knee or hip arthroplasties, the indications for shoulder arthroplasties are much more varied and range from rheumatoid arthritis, degenerative arthroses and osteonecrosis to posttraumatic osteoarthritis or osteoarthritis [3,4]. Due to the increasing demand for total endoprostheses to the shoulder, the revision of the glenoid is becoming increasingly important and demonstrates the need for basic research and development in this field.

Problems with arthroplasty or reimplantation of glenoid prostheses consist of the large-volume combined with central-peripheral glenoid defects. Due to the destroyed glenoid with bony defects, immediate insertion of a prosthesis is not possible because primary stability cannot be guaranteed by anchoring the prosthesis. So far, there have been several attempts to correct the bony defects with augmentations, however with moderate success [5,6,7,8,9,10,11,12].

A common problem was identified in connection with the screws used to fix the augmentation material. In one study, complications were reported in 78% of the observed cases of screw fracture, bent screws or metallic abrasion [13]. The main reason given for performing revision surgery is loosening of the glenoid prosthesis in 32% to 39% of cases [14,15,16], followed by instability in 23% to 30% and periprosthetic fracture in 11% of cases [14,17]. The success rate over shorter periods of time (<5 years) is given as up to 98% to 99%, which drops drastically to between 33% and 51.5% with a follow-up of >10 years [18,19,20,21]. Eccentric loads and the resulting high micro-movements at the boundary between bone cement and cortical and trabecular bone play a major role in loosening the glenoid component. Over a longer period, a phenomenon occurs which is referred to in pertinent literature as the “Rocking Horse Phenomenon” [22,23] and can subsequently lead to bone resorption and massive bony defects in the area of glenoid anchorage. When the glenoid is loosened, patients often suffer increasingly from load-dependent pain after an interval that is sometimes very long and symptom-free [24]. In the case of a confirmed loosening of the glenoid prosthesis, there is in principle an indication for a one-stage changes of the glenoid component, provided that stable anchoring is possible. However, the biggest problems with revisions of the glenoid with a new prosthesis (implant) are large bone defects or deficits in which both the cancellous part and the cortical layer are affected [25]. In these cases, insertion of a new glenoid component is often not possible because it cannot be sufficiently attached to the scapula and glenoid.

In addition to other options for augmentation with calcium phosphates, bio-glass, hydrogels, human bone allografts and biocomposites made by bioactive elements [26], there are some experimental studies about cell-instructive bioengineering procedures to support and restore preexisting bone repair and osseointegration [27,28]. An option that is commonly used as a standard is still a two-stage procedure by means of building-up the defective glenoid with autologous bony augmentations [5,6,7,10,11,29,30]. The defective glenoid component is removed, and large-volume bone deficits are built up with cortigospongious chips from the iliac crest.

In the case of extensive defects and thus large augmentations, it is necessary to fix the augmentation with screws. After sufficient osseous integration of the cortigospongious augmentation material in the glenoid (about 3 months), a new glenoid component is inserted into the bone material [31,32]. A retrospective examination of 16 revision procedures of shoulder arthroplasties with glenoid build-up showed a settlement or atrophy of the augmentation in all cases [25]. The observed shrinkage is 5 mm in 3 patients, 5 to 10 mm in six patients and more than 10 mm in two patients. All four cases of the investigated group in which the augmentation was fixed with screws showed such a large shrinkage in which the screws were exposed and touched the metallic humeral head of the humeral prosthesis. In one case, the screw even broke due to the additional load.

In cases where the screws used to fix the augmentation material comes into contact with the metallic humeral head, abrasion may occur, which may subsequently lead to complications such as metallosis [33,34].

In this paper, we propose the application of temporary protection implants for the augmentation of shoulder prosthesis revision procedures. This paper describes the development of two kinds of temporary prosthesis which support the correction of bony defects and shall significantly improve the result of glenoid augmentation. This is intended to ensure a high degree of primary stability during insertion of a glenoid prosthesis, even during revision, and consequently to achieve more effective rehabilitation.

The goals of this study are as follows: to protect screw heads against direct contact with the metal joint ball of the implant (humeral head), to prevent screws from being unscrewed, to form a sliding partner during the healing phase, better “cohesion” of the bone fragments and to provide a more even force introduction and even pressure on the augmentation material (functional load). In the following sections, the material and methods used in this study are described.

## 2. Materials and Methods

GlenoProtect is a glenoid component made of a suitable material that was developed to protect the augmentation in shoulder prosthesis revision procedures. Two variants have been developed to ensure the above-mentioned capabilities.

### 2.1. Variant 1: Multidirectional Angle-Stable Screw Connection (Rigid Fixation)

**Figure 1:**
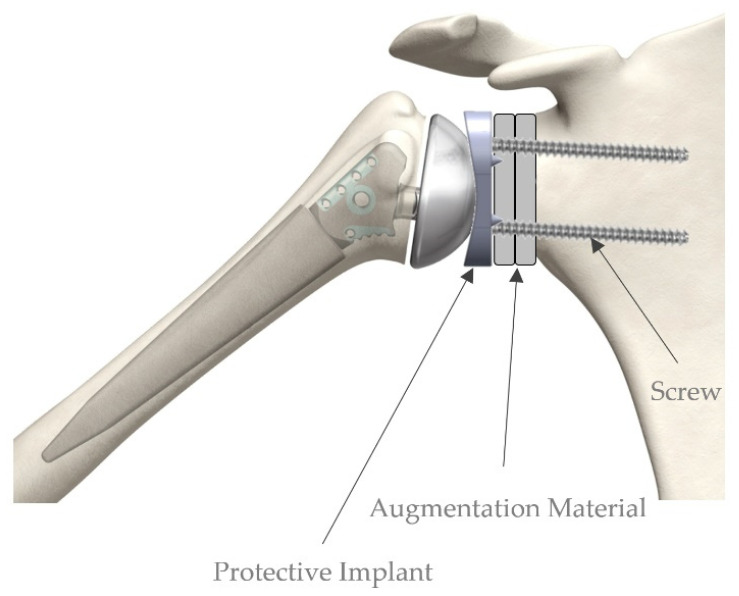
Rigid fixation of the augmentation material.

The screws have a thread in the head area and can be placed at an angle to the implant (multidirectional). To achieve angular stability, the thread cuts into the implant material and thus fixes the screw. The inclined position of the individual screws results in greater stabilization and resistance to dents (medial/proximal) and withdrawal (Figure 1).

**Figure 2:**
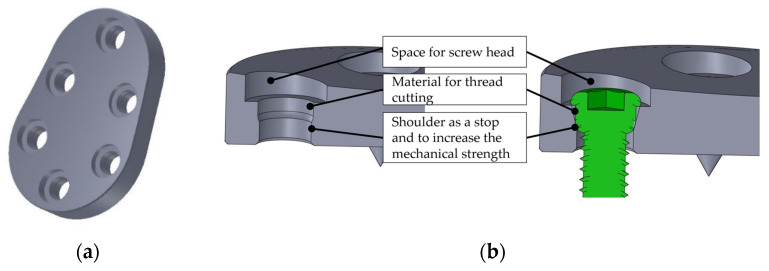
Result of detailed design of rigid variant: (**a**) three-dimensional view; (**b**) sectional view.

Resorption is the greatest unknown factor despite the protection of the augmentation. The resorption leads to a reduction of the functional pressure on the augmentation material and possibly to an increased resorption. The original design was adapted and improved in detail in several processing and optimization steps, with the aim of maximizing mechanical stability while reducing the profile cross-section (thickness or height of the implant). For this purpose, the originally designed curved oblong holes for screw connection were replaced by circular holes, as the intended cutting of the thread of the screw heads did not provide sufficient stability in this case. In addition, the radius of the implant surface was adjusted to achieve an even lower implant height. The result of the detailed design can be seen in Figure 2.

**Figure 3:**
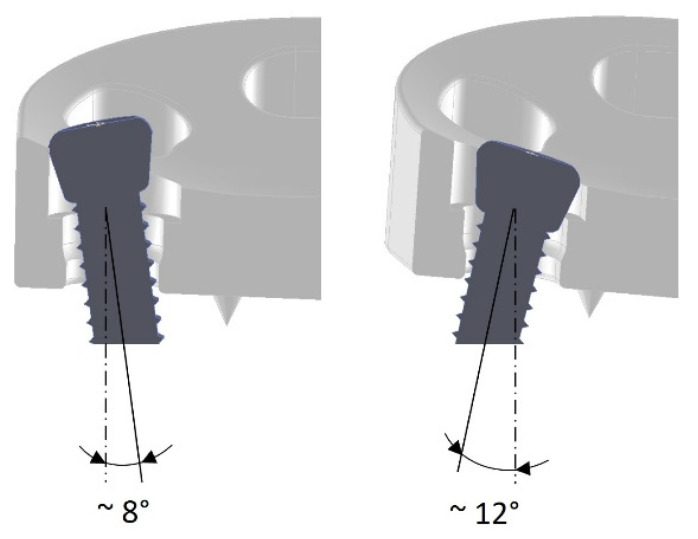
Multidirectional screw positioning possibility.

Particular attention was paid to the design of the fixing holes. The exact geometry of these holes is important to ensure that the screw heads are held securely and that the screw heads do not project beyond the implant surface. The implant or the fixing holes were designed for the use of osteosynthesis screws “Locking Screw 3.5 mm” from the Small Fragment Locking Compression Plate (LCP) System (DePuy Synthes Companies, Zuchwil, Switzerland, and Warsaw, IN, USA, https://www.jnjmedicaldevices.com/en-EMEA/companies/depuy-synthes, accessed on 8 July 2022). The screws can be introduced in multiple directions, i.e., at different angles to each other, in order to adapt to the respective anatomical situations and to increase the mechanical stability of the fixation (Figure 3).

### 2.2. Variant 2: Dynamic Fixation (Angular Stable Pins)

**Figure 4:**
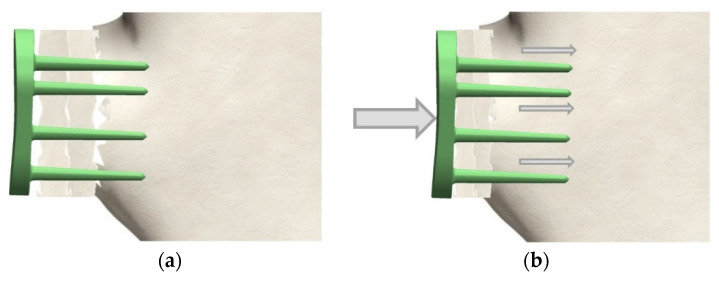
Dynamic fixation using angle-stable pins: (**a**) initial post-operative situation; (**b**) dynamic load results in compression and functional stimulus of the augmented bone.

**Figure 5:**
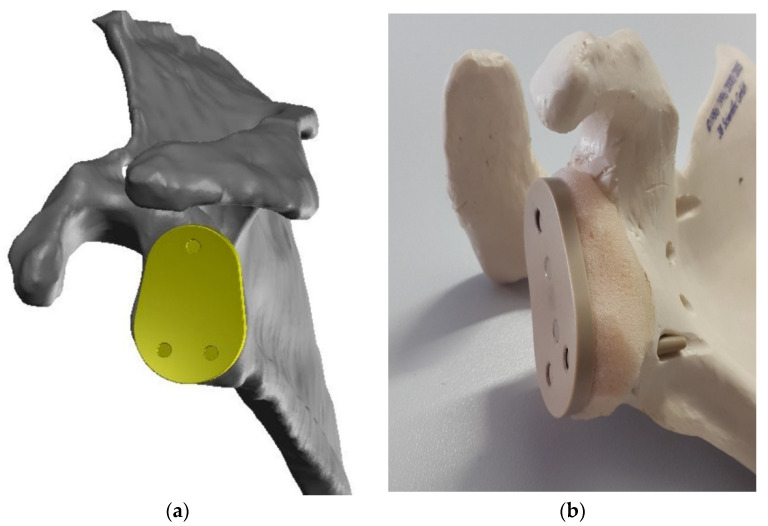
Visualization of dynamic variant of GlenoProtect on glenoid/scapula (**a**) digital model for simulation purpose (bone defect, augmentation not shown) and (**b**) analog model for development purpose (foam material simulates the bone augmentation).

To compensate for the risk of functional underloading of the augmentation material, a dynamic system is proposed. Instead of screws, pins are used which only have a thread in the head area that cuts into the implant material. The pins should be placed at a right angel to the implant backside (defined by the geometry), as shown in Figure 4 and Figure 5.

Only shear and torsional forces in the medial or sagittal plane are absorbed. The entire unit can sink if the bone block atrophies and the dynamic load and thus a functional stimulus is maintained. This is to reduce bone resorption. The dynamic version can be fitted with up to five pins, and the most appropriate pins should be used depending on the specific circumstances.

As an implant material, the use of a plastic material should be considered as the most sensible option. Metallic materials are critical due to the sliding pairing with the also metallic humeral head [35,36]. PE (polyethylene) and PEEK (polyetheretherketone) are possible plastics [37,38,39]. PE is the most commonly used material for primary glenoid components, where ultra-high molecular weight polyethylene (UHMWPE) is used. PE has good sliding properties, is easy to process and is comparatively inexpensive. With PEEK, the raw material is more expensive, however, it has more suitable mechanical properties than PE. Thus, the modulus of elasticity is closer to that of bone. This results in a better or natural distribution of force on the bone [40,41]. PEEK is a high-performance biomaterial suitable for long-term implantation. It is used to manufacture a wide variety of medical devices and human implants (dentistry, orthopedics and traumatology) and is used in a variety of ways in these applications [42,43,44,45]. Due to its chemical composition, PEEK is a very pure and inert material. Extensive biocompatibility tests do not provide any evidence of cytotoxicity, systemic toxicity, irritation or acroscopic reactions [31,32,33,34,35]. In addition, the very low levels of residues and extractable metal ions minimize the potential risk of allergic reactions commonly associated with nickel or other metal ions. In addition, PEEK can be sterilized using all common methods. PEEK is suitable for gamma, ethylene oxide and saturated steam sterilization [46].

In order to determine the mechanical strength, corresponding simulations were carried out. The selected implant variants were analyzed using FEM (Finite Element Method), also known as FEA (Finite Element Analysis) [47]. The simulation program Abaqus (Dassault Systèmes, Vélizy-Villacoublay, France, http://www.3ds.com, accessed on 8 July 2022) was used. A material approved for medical applications in the human body and repeatedly proven in implants is PEEK-Optima from Invibio Ltd. (for properties, see Table 1: Material properties PEEK, Invibio Ltd., Lancashire, UK). These material properties were used as a basis for the FEM analysis.

The load and force assumptions for the simulation were selected by Westerhoff and Bergmann [48] according to the measurements at the Julius Wolff Institute. For the FEM analysis, the data set “Lifting a weight of 10 kg” was used, since the measurement results here show the highest values. The data give a maximum value of 1500 N for the force acting normally on the implant surface. After consultation with shoulder surgeons, a maximum realistic force of 500 N is to be assumed for the intended application. The simulation was performed with 1500 N as well as with 500 N. In the FEM simulation, static forces/loads are applied and the resulting stresses, displacements (elastic) or plastic deformations are determined. For this purpose, a so-called substitute model must be created, which represents a section of the entire situation, reflecting the relevant force application. For the calculation of the stresses in the protective implant, the replacement model was in the form of a hemisphere representing the humeral head with an equally distributed load application.

**Figure 6:**
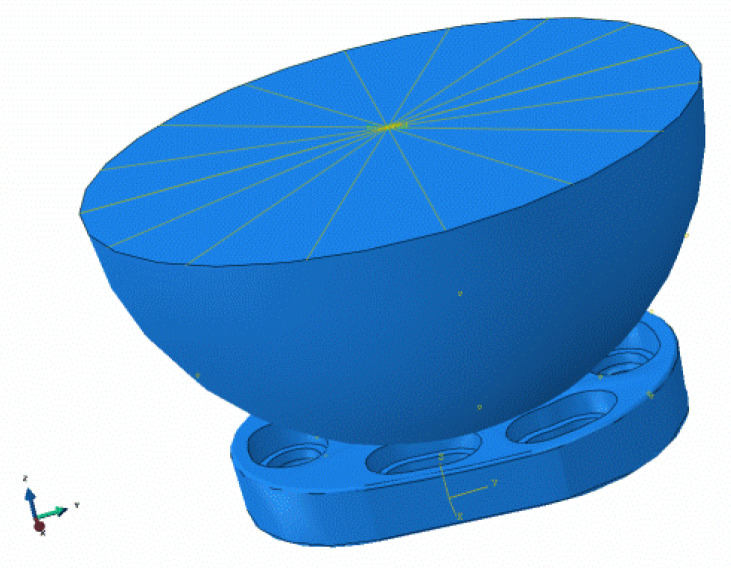
Modeling of the load for the FEM analysis.

For the simulation of the rigid version, a joint ball made of stainless steel, with a diameter of 48 mm, and its support centered on the implant was modelled (Figure 6).

Fixation of the implant was assumed with four screws. The posterior implant surface was assumed without bone support, so the implant is only held in place by the screws. Thus, the determined displacement (corresponds to “deflection”) of the implant can act as a stimulus on the augmentation. At the simulation model for the dynamic variant, the joint ball is resting eccentrically on the implant. The fixation of the implant was assumed at the end of the conical pins. The eccentric loading and clamping of the pin ends result in a displacement and thus a (rotational) moment at the connection points of the pins with the implant main body. This in turn represents the load situation that occurs when the implant is attached to the bone via the pins and “tilts” the implant surface away due to an eccentric load (with corresponding movement of the shoulder).

Mechanical testing was performed in order to confirm the results from the simulation and to demonstrate the effective strength of the implant variants and the respective fixing methods. For the mechanical tests, a specific test stand was built at the Center for Medical Physics and Biomedical Engineering of the Medical University of Vienna at the AKH Vienna.

The test bench is used to simulate the movements of the shoulder joint. For this purpose, the test stand was primarily designed as an actuator-controlled pendulum system. Thus, it is possible to perform cyclic movements of a simulated humeral head prosthesis in relation to the glenoid protective implant. The contact force (force normal to the implant surface) and the type of movement of the ball of the humeral head (from rolling on the implant surface to a pure friction movement at one point) are adjustable.

The first mechanical test is intended to confirm the mechanical stability of the combination of the implant itself and the fastening elements (screws in the rigid and pins in the dynamic variant) under static loading. The implant itself as well as the fastening elements and the connection of the implant with the fastening elements must be sufficiently strong. In particular, the connection of the implant with the screws or pins represents a critical point. With a static implant, the screw heads must not protrude beyond the implant surface under load.

Following the static load test, a dynamic load test was carried out. For this purpose, the pendulum frame was oscillated with an angular deflection of ±30°. Due to the dynamics of the system and the centrifugal forces that occur when the pendulum frame is loaded with the additional weight, the test was carried out with a maximum load of 1000 N.

Comparative measurements were carried out to determine differences in the abrasion behavior of different materials. The following test materials were examined in Table 2:

To create largely real conditions, the test objects were placed in a shell filled with a physiological solution of H_2_O, agar-agar and NaCl during the pendulum motion. Agar-agar was added to increase the viscosity of the liquid, as the synovial liquid is also more viscous.

A high-precision galvo scanner (scanner galvanometer) was used for the tactile measurement of the surface [49] to quantitatively determine the abrasion. The galvo scanner has a resolution of 200 nm. This means that any abrasion in the form of “material shrinkage” can be measured at the relevant point. This method does not determine the abrasion as detached particles, but whether there is abrasion on the implant surface. These depressions in the material can be very precisely measured with the galvo scanner and are thus displayed quantitatively.

**Figure 7:**
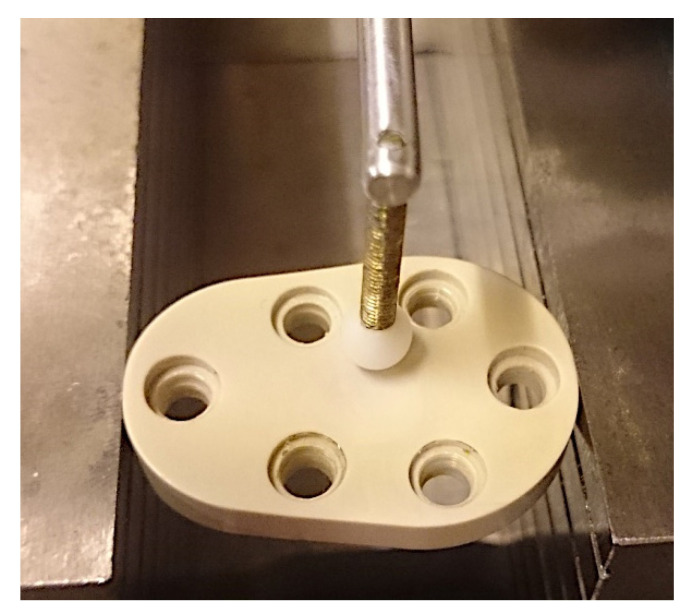
Measuring tip of the galvo scanner for tactile surface measurement.

The test objects were clamped on a cross table. The galvo scanner was fixed via a 3D articulated tripod so that its lever arm rests on the test object (Figure 7). Now the cross table was moved manually, and the output signal was recorded with a DAQ measuring system (DEWE-43 from DEWESoft, Trobvlje, Slovenia, http://www.dewesoft.com, accessed on 8 July 2022).

## 3. Results

In this chapter, the results for the simulation (FEM), mechanical testing results and the abrasion measurements are summarized.

### 3.1. Simulation

The results of the FEM simulation for both implant variants are presented in Table 3.

**Figure 8:**
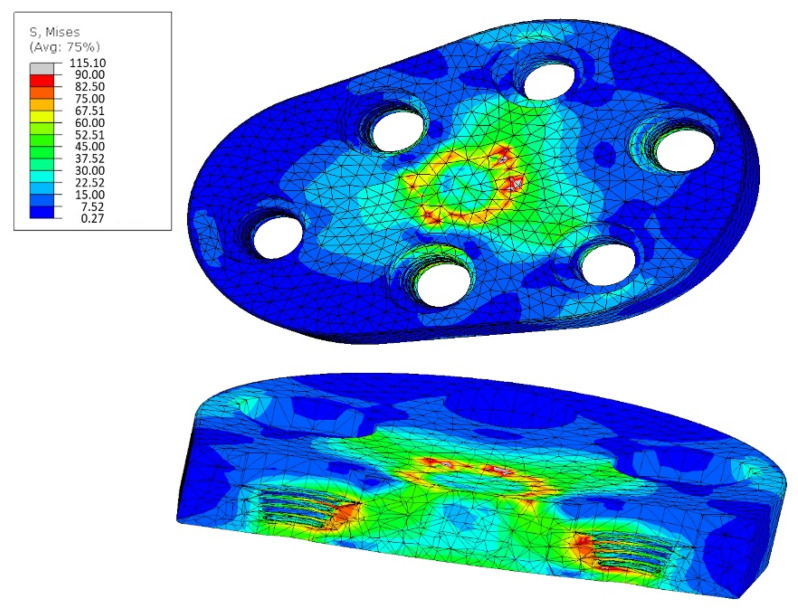
FEM Analysis of the rigid variant: strain at 1500 N, scaling 90 MPa.

With the rigid variant at 1500 N load, stresses greater than 90–100 MPa occur selectively in the material (Figure 8).

Since these areas are rather small and surrounded by areas of significantly lower stress, it can be assumed that structural integrity will be maintained. However, it is to be expected that superficial damage may occur (the areas of high tension lie on the surface of the implant in contact with the joint ball).

**Figure 9:**
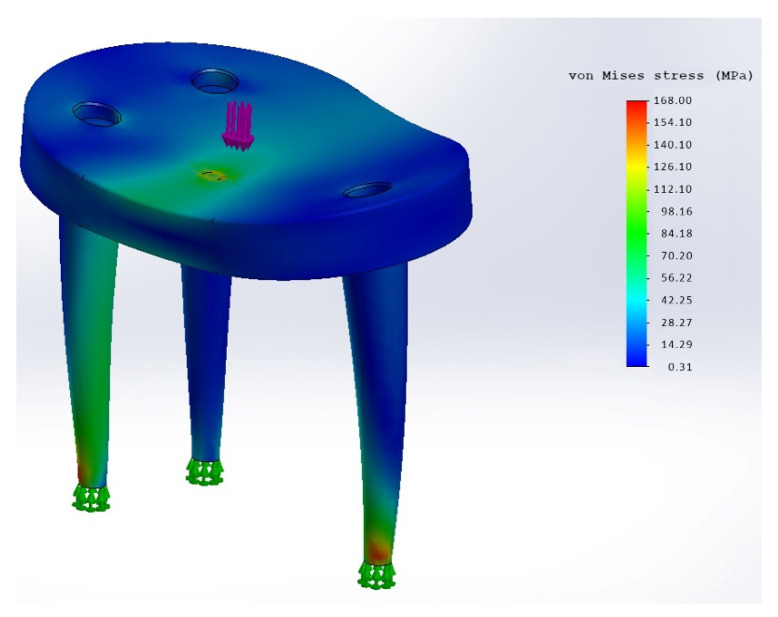
FEM Analysis of the Dynamic variant: strain at 500 N, scaling 168 MPa.

Peak strain values of up to 168 MPa occur selectively in the material of the dynamic variant (see Figure 9).

However, these areas are very limited and are justified by the fact that the ends of the pins were firmly clamped for the simulation. This is exactly where these high stresses occur. It is more realistic to distribute the forces over a larger surface of the pins. The maximum strain values occurring over a large area are in the range of 100 MPa. However, this is already at the limits of the material.

The simulation with a load of 1500 N resulted in peak strain values well above 100 MPa. This would mean that the implant would no longer be able to withstand the load. The high stresses occur again in isolated areas and could occur due to the modeling (clamping only at the tip of the conical pins would distribute the dissipation of forces along the pins).

Summary:

The results of the FEM analysis of the rigid variant show that there will be no permanent damage (plastic deformation) when the load is 500 N. Under a load of 1500 N, plastic deformations or material damage can occur on the contact surface with the joint ball, but the overall strength of the implant and the screw connections would still be guaranteed.

The results of the FEM analysis of the dynamic implant variant show that loads of 500 N do not lead to any damage to the implant or that the entire structural integrity is preserved. At a load of 1500 N, the maximum permissible stresses in the material would be significantly exceeded. In such a case, modeling becomes very difficult, and it is very likely that the resulting stresses arise due to the model assumptions. However, since a maximum of 500 N can be assumed as a realistic force, the simulation of the dynamic implant variant also shows that it has sufficient mechanical stability.

### 3.2. Mechanical Testing

#### 3.2.1. Rigid Variant

**Figure 10:**
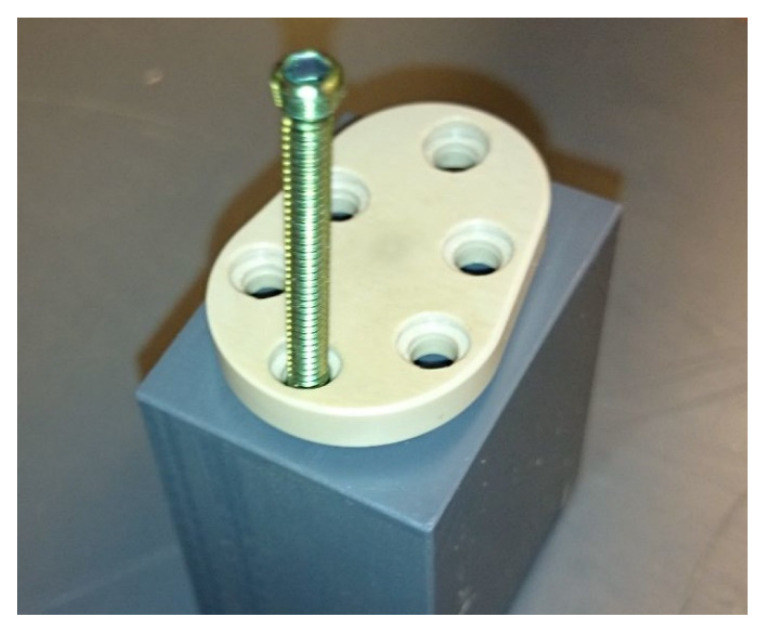
Fixing the test implant at the test block (represented is only one screw before insertion, the test implant was fixed with three screws).

For the test, the protective glenoid implant was attached at a distance from a test block with three angular stable osteosynthesis screws (Figure 10). This simulates the case where the protective glenoid implant was attached to the scapula above the graft and the graft was already slightly resorbed. This creates a small gap and the forces applied to the implant are transmitted exclusively via the screws into the scapula, which is the worst case from a mechanical point of view. In this case, the force is transmitted in the area of the small thread onto the head of the osteosynthesis screws. The screw heads must remain securely fixed in the implant and not “tear out” so that the screw heads protrude beyond the implant surface.

The test implant was loaded with 1300 N. There was no damage to the implant and the threaded connections also withstood the load. This result corresponds to the behavior expected from the FEM simulation (no permanent deformation, maintenance of overall stability up to 1500 N).

The implant and the threaded connection were also stable in the test with dynamic loads.

#### 3.2.2. Dynamic Variant

**Figure 11:**
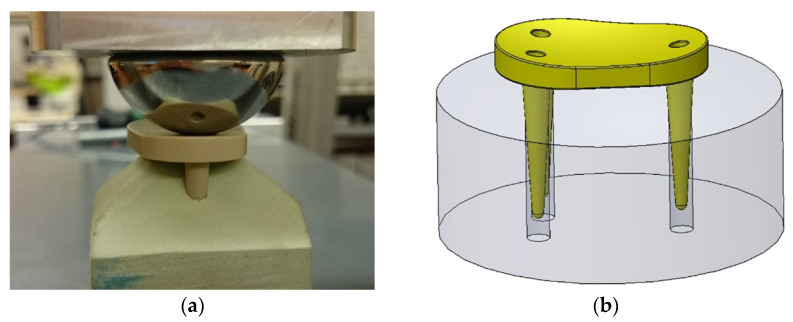
Test abutment for the dynamic implant variant: (**a**) view of the actual test setup; (**b**) schematic view of the test block.

The dynamic implant variant with the conical spikes for fixation, which allow the implant to sink, was also fixed in a test block. This test block has a convex surface and parallel holes to accommodate the fixing pins; see Figure 11.

The test implant was loaded in the same way as in the static loading test of the rigid implant variant. The load did not cause any mechanical damage to the test implant.

The dynamic test implant was also tested with dynamic loads, where it was attached to the test block and the pendulum frame was made to vibrate. This resulted in a fracture of the conical pins in the area of the thread transition. However, the fracture did not occur until a forced extreme load was applied, with the force being applied eccentrically and transversely (pivot point of the joint ball outside the central axis). Such loads are not to be expected under real conditions, so this test also confirmed the stability of the dynamic implant variant.

### 3.3. Abrasion Measurement

**Figure 12:**
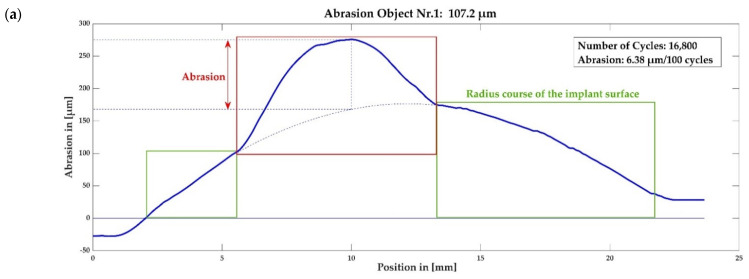

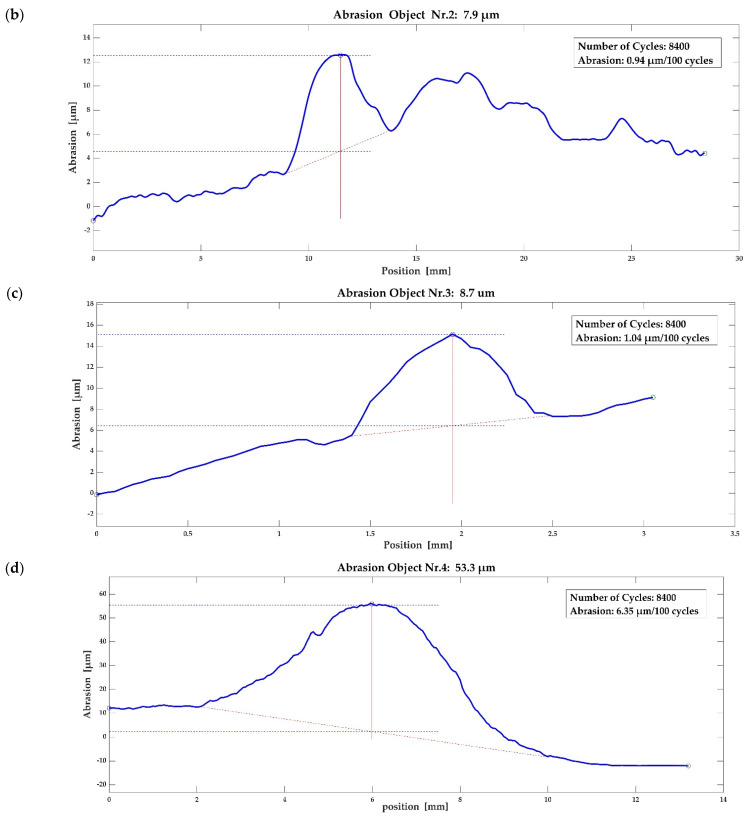
Abrasion measurement curves: (**a**) test object 1, including additional description of the sections of the curve; (**b**) test object 2; (**c**) test object 3; (**d**) test object 4.

The summary of all abrasion measurement results is shown in Figure 12a–d. The different diagrams show the measurement curves of test objects 1–4 (compare Table 2). Figure 12a contains additional descriptive elements.

The position along the implant surface (geometric longitudinal axis) is plotted on the x-axis and the deflection (normal distance) of the galvo scanner measuring tip is plotted on the y-axis. The y-axis thus represents the measured abrasion. A standardized abrasion value in µm/100 cycles is exhibited for each object. Sudden, strongly deviating signals correspond to depressions in the surface and are a direct measure of the abrasion occurring at this point.

The results clearly show that the abrasion to be expected with the glenoid protective prosthesis is much lower than with a standard glenoid component of an anatomical shoulder prosthesis made of PE.

This result corresponds to the order of magnitude of a study already carried out, in which a wear factor was determined that is 10 times higher for a friction pairing of UHMWPE with CoCrMo steel than for PEEK with CoCrMo steel [50], as compared in Table 4.

The difference between the result with the technical PEEK (test object 1) and the medical grade PEEK (test objects 2 and 3) can be explained by different mechanical properties due to the use of different starting materials for the synthesis. The technical PEEK used for the test has a notched impact strength of 3.5 kJ/m^2^, whereas the medical grade PEEK has a notched impact strength of 5.5 kJ/m^2^.

## 4. Discussion

Within the scope of the present paper, two variants of a glenoid protective prosthesis were developed, with the following functions in the foreground:

Protection of the screw heads against direct contact with the joint ball of the metal humeral head prosthesis, prevention of unscrewing or loosening of the screws, formation of a sliding partner during the healing phase, better “holding together” of the bone fragments as well as targeted application of force and uniform pressure on the augmentation material (functional load).

The development was carried out based on specific known problems with the method currently used and potential improvements based on the selected design. The results are prototypes, which were first validated in mechanical tests and then tested in a clinical pilot study. These two variants were developed for the research program for prosthetics, biomechanics and biomaterials research at Paracelsus Medical University, in order to take the second question into account and to enable a direct comparison within the framework of a clinical study. The specific question is whether significantly better results can be achieved with a dynamic system of fixation of the protective prosthesis or the augmentation compared to a rigid fixation. From a biomechanical point of view, it was postulated that the atrophy of the augmentation should be lower with dynamic fixation. This was justified accordingly in the presented research carried out.

Both implant variants were tested for stability and strength by means of FEM simulation. In addition, based on the results of the FEM simulation, it can be assumed that the rigid design of the protective denture also has advantages in terms of osseointegration of the augmentation, since the modulus of elasticity of the implant material used (PEEK) is similar to that of cortical bone tissue and thus exerts a natural load on the augmentation, which in turn is intended to reduce atrophy.

Final mechanical tests confirmed the results previously obtained in the FEM simulations regarding the stability of both implant variants. In addition to checking the stability, the abrasion behavior was also measured during the mechanical tests, since in contrast to the surface loading in a total prosthesis with a form-fitting ball and ball socket, the present protective implant with only a slight concave curvature is subjected to a theoretical point load. Despite the compressibility and elasticity of the material, the contact surface is relatively small, so that an experimental test of the abrasion properties is necessary. Here, too, the result was positive to the extent that the abrasion determined was very low (significantly less than with the material of a commercial glenoid prosthesis measured in comparison).

In addition to the actual development of the glenoid protection prostheses, appropriate documentation was carried out to register and conduct a clinical study to test the implants. The documentation has also been prepared in accordance with applicable standards and guidelines for the development of a medical device in order to facilitate possible approval and marketing.

As mentioned above, a clinical pilot study will be carried out following the discussed activities with the results obtained. The purpose of this study is to demonstrate that the use of a glenoid protective prosthesis can significantly improve the outcome of glenoid revision in shoulder prostheses. Depending on the findings of this study, various further developments would be conceivable. A promising option would be to not solely use a protective prosthesis during the healing period of the augmentation material (still two-stage procedure): The old glenoid component is removed and the glenoid is rebuilt using the protective prosthesis, followed by the insertion of the new glenoid component in a second surgery, but, in addition, a “revision glenoid prosthesis” is inserted which remains permanently implanted.

The theoretical background and the basics for it were determined and corresponding potential suggestions for improvement were implemented in the form of a glenoid protective prosthesis. “In silico” (FEM simulations) investigations and mechanical tests on prototypes served to verify the (bio)mechanical properties of the protective prostheses. A particularly interesting result was the abrasion measurements. It was found that the abrasion of the protective implants made of PEEK is significantly lower than that of the glenoid component of a standard anatomical shoulder prosthesis. This fact opens up the possibility of further developing the proposed protective prosthesis in such a way that it could be used as a permanent revision implant, thus avoiding the need for a second intervention.

Two variants were designed in the development of the protective prosthesis, and the subsequent clinical trial will show whether the dynamic variant has the postulated advantages over the rigid variant.

## Figures and Tables

**Table 1:** Material properties PEEK.

Property	Units	PEEK-OPTIMA
Tensile Strength (Yield)	MPa (ksi)	115
Tensile Elongation (Break)	%	20
Flexural Modulus	GPa	4
Flexural Strength	MPa	170
Izod Impact (Unnotched)	kJm^−2^	Does not break
Izod Impact (Notched)	kJm^−2^	4.7

**Table 2:** Overview of test objects for abrasion measurement.

Test Object No.	Material
Test object 1	Test sample of a glenoid protective prosthesis made of technical PEEK: KETRON PEEK-1000 (not medical grade)
Test object 2	Test plates made of medical grade PEEK No. 1
Test object 3	Test plates made of medical grade PEEK No. 2
Test object 4	Comparison sample of a glenoid component of an anatomical shoulder prosthesis (Global Advantage Keeled Glenoid, DePuy) made of PE (1020 XLK UHMWPE)

**Table 3:** Summary of FEM simulation results.

Tensile Elongation	Rigid Variant	Dynamic Variant
max. Tensile Elongation PEEK-Optima	20%	20%
Analysis Sample Material	24%	24%
Result FEM (500 N)	2.2%	-
**Strain**	**Rigid Variant**	**Dynamic Variant**
Tensile Strength PEEK-Optima	100 MPa	100 MPa
Analysis Sample Material	117 MPa	117 MPa
Max. Strain FEM (500 N)	60 MPa	~100 MPa (168 MPa peak)
Max. Strain FEM (1500 N)	90 MPa	>100 MPa
**Displacement**	**Rigid Variant**	**Dynamic Variant**
Max. Displacement (deflection/sag) at 500 N FEM	0.1 mm	0.95 mm

**Table 4:** Total Wear Factors (values times 10^−6^ mm³/Nm) for Carbon Fiber-Reinforced PEEK-OPTIMA against different Counterparts and UHMWPE against CoCrMo steel [47].

CFR PEEK-OPTIMA/CFR PEEK-OPTIMA	CFR PEEK-OPTIMA/Alumina	CFR PEEK-OPTIMA/CoCrMo Steel	UHMWPE/CoCrMo Steel
0.34	0.18	0.1	1.1
